# The ESAS-score: A histological severity grading system of subarachnoid hemorrhage using the modified double hemorrhage model in rats

**DOI:** 10.1371/journal.pone.0227349

**Published:** 2020-02-25

**Authors:** Dorothee Mielke, Kim Bleuel, Christine Stadelmann, Veit Rohde, Vesna Malinova

**Affiliations:** 1 Department of Neurosurgery, Georg-August-University Göttingen, Göttingen, Germany; 2 Department of Neuropathology, Georg-August-University Göttingen, Göttingen, Germany; University Hospital Basel, SWITZERLAND

## Abstract

**Objective:**

The amount of extravasated blood is an established surrogate marker for subarachnoid hemorrhage (SAH) severity, which varies in different experimental SAH (eSAH) models. A comprehensive eSAH grading system would allow a more reliable correlation of outcome parameters with SAH severity. The aim of this study was to define a severity score for eSAH related to the Fisher-Score in humans.

**Material and methods:**

SAH was induced in 135 male rats using the modified double hemorrhage model. A sham group included 8 rats, in which saline solution instead of blood was injected. Histological analysis with HE(hematoxylin-eosin)-staining for the visualization of blood was performed in all rats on day 5. The amount and distribution of blood within the subarachnoid space and ventricles (IVH) was analyzed.

**Results:**

The mortality rate was 49.6% (71/143). In all except five SAH rats, blood was visible within the subarachnoid space. As expected, no blood was detected in the sham group. The following eSAH severity score was established (ESAS-score): grade I: no SAH visible; grade II: local or diffuse thin SAH, no IVH; grade III: diffuse / thick layers of blood, no IVH; grade IV: additional IVH. Grade I was seen in five rats (7.9%), grade II in 28.6% (18/63), grade III in 41.3% (26/63) and grade IV in 22.2% (14/63) of the rats with eSAH.

**Conclusion:**

The double hemorrhage model allows the induction of a high grade SAH in more than 60% of the rats, making it suitable for the evaluation of outcome parameters in severe SAH.

## Introduction

In patients with aneurysmal subarachnoid hemorrhage (SAH), the severity of the bleeding is related to the amount of extravasated blood. The amount of extravasated blood is graded by the Fisher grading system. The SAH severity plays an essential role in the prognosis, and the Fisher grade can be considered as a prognostic parameter. Many aspects of the pathophysiological process after SAH are not yet fully understood. Thus, animal experiments are essential for improving our pathophysiological understanding of SAH. Today, mostly rodents were used for induction of experimental SAH (eSAH). So far, different SAH-models have been described in rodents [1–13]. In order to allow a more reliable correlation of outcome parameters with the eSAH severity and better comparability of SAH-models, the definition of a comprehensive grading system for the severity of an eSAH is useful. Sugawara et al. have described an eSAH grading system, based on a macroscopic semiquantitative evaluation of the amount of subarachnoid blood after sacrificing the animals, 24 hours following the SAH induction using the perforation model in rats [11]. Although this grading system allows a reliable evaluation of eSAH severity, it is not applicable in experimental studies with an expected duration of more than 24 hours, which are required for all investigations focussing on delayed ischemic complications. The depiction of blood within the subarachnoid space using conventional imaging techniques such as magnetic resonance imaging (MRI) and computed tomography in small animals like rodents is challenging. A grading system for eSAH based on 7 Tesla MRI was already described using the endovascular perforation model in mice and rats [14–16]. However, a high field MRI is not available in the majority of research centers. No grading system for the evaluation of eSAH-severity applying blood injection eSAH-models in rats has been defined so far. In this study, we aimed to define an easy to apply eSAH severity grading scale based on post mortem histological analysis five days after SAH induction and evaluated whether the modified double blood injection eSAH-model can reliably induce a severe SAH in rats.

## Material and methods

### Ethical statement

The article was written according to the ARRIVE guidelines [17]. All experiments were conducted in accordance with the “Guide for the Care and Use of Laboratory Animals of the NIH”and were ethically approved by the Government of Lower Saxony (AZ 13/1055). An expected mortality rate of approximately 50% was stated in the protocol and was approved by the Government of Lower Saxony. The euthanasia of the animals was performed under general anaesthesia by transcarcial perfusion at the end of the experiment on day 5. Seventy one animals died directly after the blood injection either on day 1 or day 2 without euthanasia. The reason for death was a severe SAH directly leading to death. All surgical steps were performed under general anaesthesia, and all efforts were made to minimize suffering.

### Experimental setting

The experiments were performed on 143 male Sprague-Dawley rats (Charles River, Germany), whereof 135 rats were in the SAH-group and 8 rats in the Sham-group. The sample size was calculated considering the reported mortality rate of 50% in the double blood injection model [18].

The rats were assigned to the SAH-group or the Sham-group according to the random principle. The animals were housed in a temperature- and humidity-controlled room on a 12-hour light / 12-hour dark cycle in single cages under standard laboratory conditions, with food and water ad libitum. All rats were delivered with a body weight of 200 g and had one week of accommodation time before the experiments started. The duration of the experiment was 5 days: on day 1 and day 2 the blood was injected; on day 3, day 4 and day 5 a neurological evaluation of the rats was performed using a pre-defined neuroscore for the assessment of motor function in rats [19].

Additionally, the righting reflex was assessed. The neurological evaluation was performed by a veterinarian, who was blinded to the assignment of the animals to the SAH-group or the Sham-group. On day 5, transcardial perfusion was performed and the brains were removed for the histological analysis. The body weight of the rats at the beginning of the experiments ranged from 245 to 350 g (mean 304.2 g). The mean body weight was 293.7 g (range 230–380 g) on day 2, 276.8 g (range 230–345 g) on day 3, 273.4 g (range 225–328 g) on day 4 and 274.2 g (range 225–325 g) on day 5, respectively. A weight loss of > 20% of the initial body weight within 24 hours was defined as a termination criterion, which was not reached by any of the included animals in this study.

### Induction of subarachnoid hemorrhage

Anesthesia was performed by intraperitoneal injection of an anesthetic cocktail (1 ml) consisting of 0.3 ml medetomidine (Cepetor® 1 mg/ml) and 0.7 ml ketamine (Ketamin® 10% Solution) with an applied dosage of 0.1 ml per 100 g body weight. Since this anesthesia does not compromise the respiration, intubation of the rats was not necessary. During the procedure, the body temperature was maintained between 36.5 and 37.5°C by a heating blanket, controlled by a rectal temperature probe (Homeothermic Monitor, Harvard Apparatus, Hugo Sachs Elektronik, Germany). eSAH was induced in 135 Sprague Dawley male rats using the modified double hemorrhage model, as recently described by our study group and first described by Güresir et al [18,20]. This model consists of the injection of 0.25 ml autologous arterial blood in the subarachnoid space on two consecutive days. The blood was injected into the cerebellomedullary cistern. In the Sham-group, the same amount of saline solution instead of autologous arterial blood was injected. For postoperative pain control, all rats received buprenorphine (Temgesic®, RB Pharmaceuticals Limited, Berkshire, United Kingdom) (0.03–0.05 μg/kg to 0.1 μg/kg body weight s.c.) as well as 5 ml saline solution s.c. twice a day and metamizole (1.33 mg/ml drinking water p.o.) continuously. The animals were clinically examined at least three times per day by a veterinarian and buprenorphin was additionally injected if clinical signs of pain were detected.

### Histological analysis

On day 5, transcardial perfusion with phosphate buffered saline (PBS) and paraformaldehyde (4%) through a transthoracic approach was performed. After perfusion-fixation, the brains were carefully removed from the skull and were cut in 3 mm-thick coronal slices and embedded in low-melting paraffin. For histological analysis slices of 1–3 μm were cut on a microtome. HE(hematoxylin-eosin)-staining was performed in all rats according to standard local procedures. The amount and distribution of blood within the subarachnoid space and ventricular system (IVH) were analyzed according to the described grading scale. Additionally, the reaction of the meninges (cell proliferation) against the subarachnoid blood was evaluated and semiquantitatively divided into slight reaction and pronounced reaction. The histological analysis was performed multiple times by a trained doctoral student (K.B) in order to minimize intra-observer reliability. Then, the analysis was performed by an associate professor of neuropathology (C.S.) in order to allow an inter-observer reliability.

The following pre-defined criteria were applied for the differentiation of slight and pronounced meningeal reaction: if cell proliferation was only occasionally detected affecting only a small focal area of the meninges the presence of slight meningeal reaction was documented, if the cell proliferation was diffusely or multifocally distributed along the meninges, a pronounced meningeal reaction was diagnosed.

### Statistical analysis

The statistical analysis was based on descriptive statistical methods. Correlation analysis of the ESAS-score with the meningeal reaction and with the neuroscore was performed by calculation of the Spearman’s correlation coefficient. The statistical analysis was performed using the JMP statistical software (JMP Statistical Discovery^™^ From SAS Institute, Version 14.2.0).

## Results

In all, except two of the eSAH rats, blood was visible within the subarachnoid space. As expected, no blood was visible in the sham group (n = 8).

### eSAH grading

**Fig 1:**
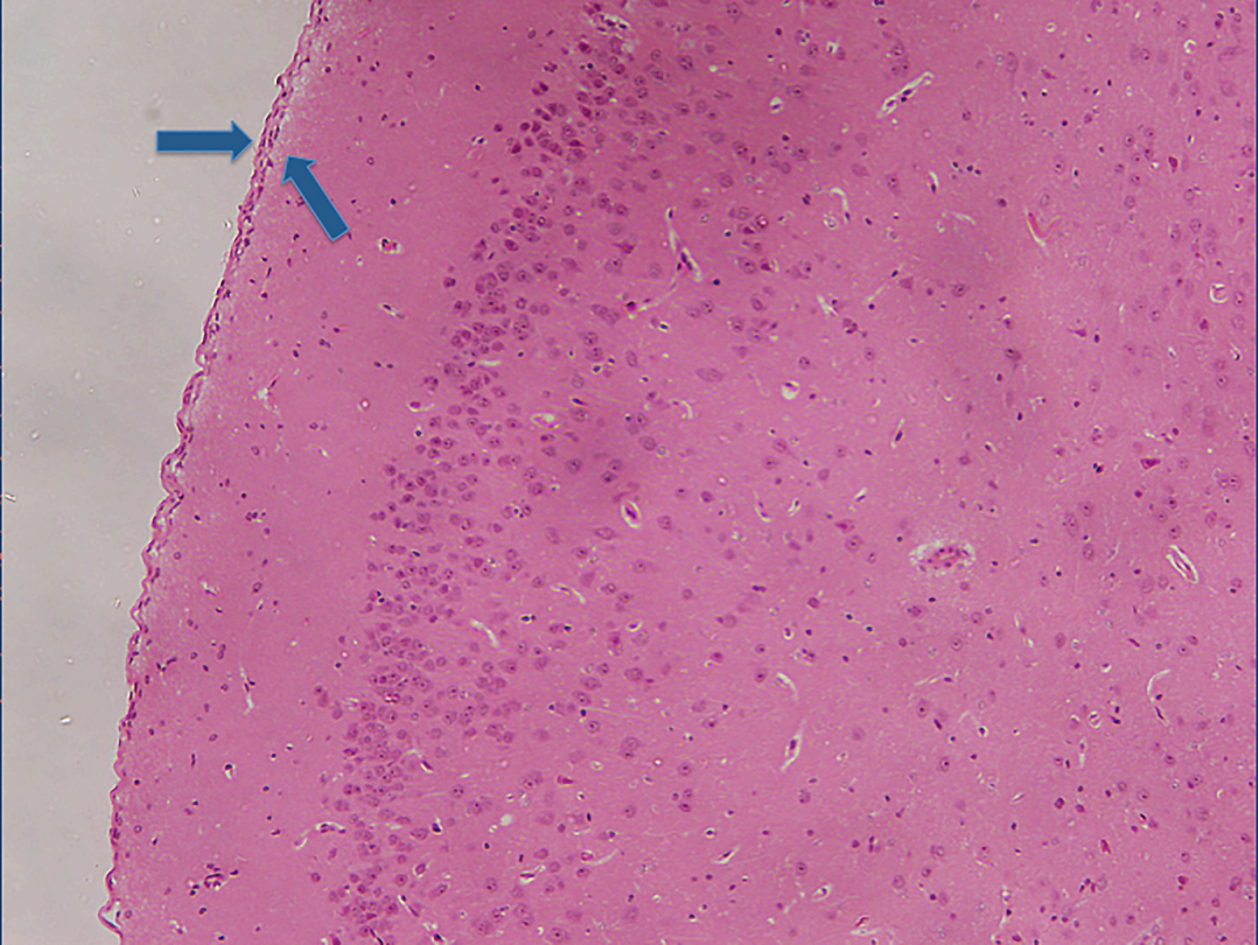
Grade I: A section of brain surface with no visible blood within the subarachnoid space (two blue arrows are marking the subarachnoid space).

**Fig 2:**
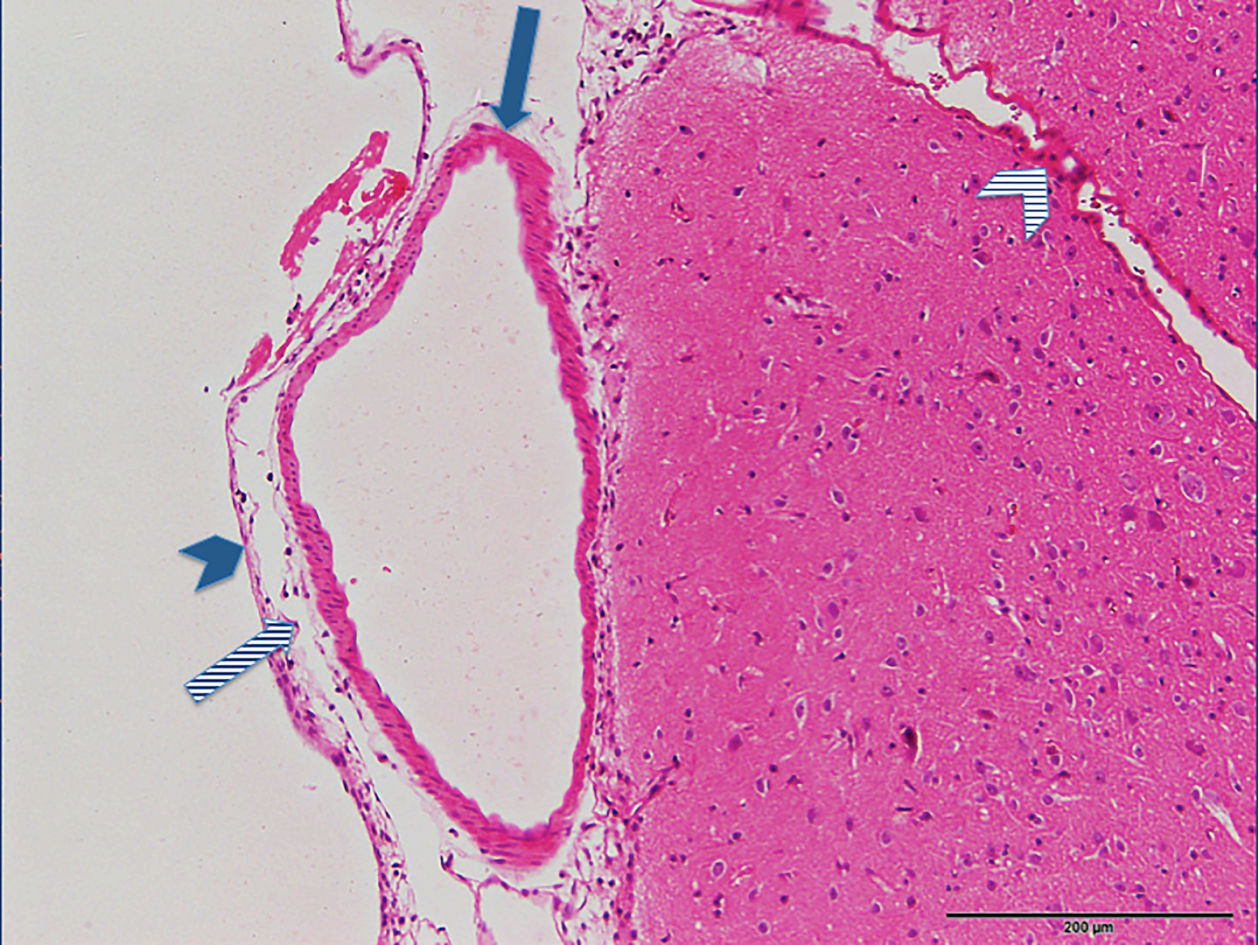
Grade II: A section of brain surface showing the surrounding subarachnoid space with a thin layer of subarachnoid blood visible within the subarachnoid space (long striped arrow pointing at the subarachnoid space, short striped arrow pointing at a thin layer of subarachnoid blood, long blue arrow pointing at a vessel within the subarachnoid space and short blue arrow pointing at the arachnoidea).

**Fig 3:**
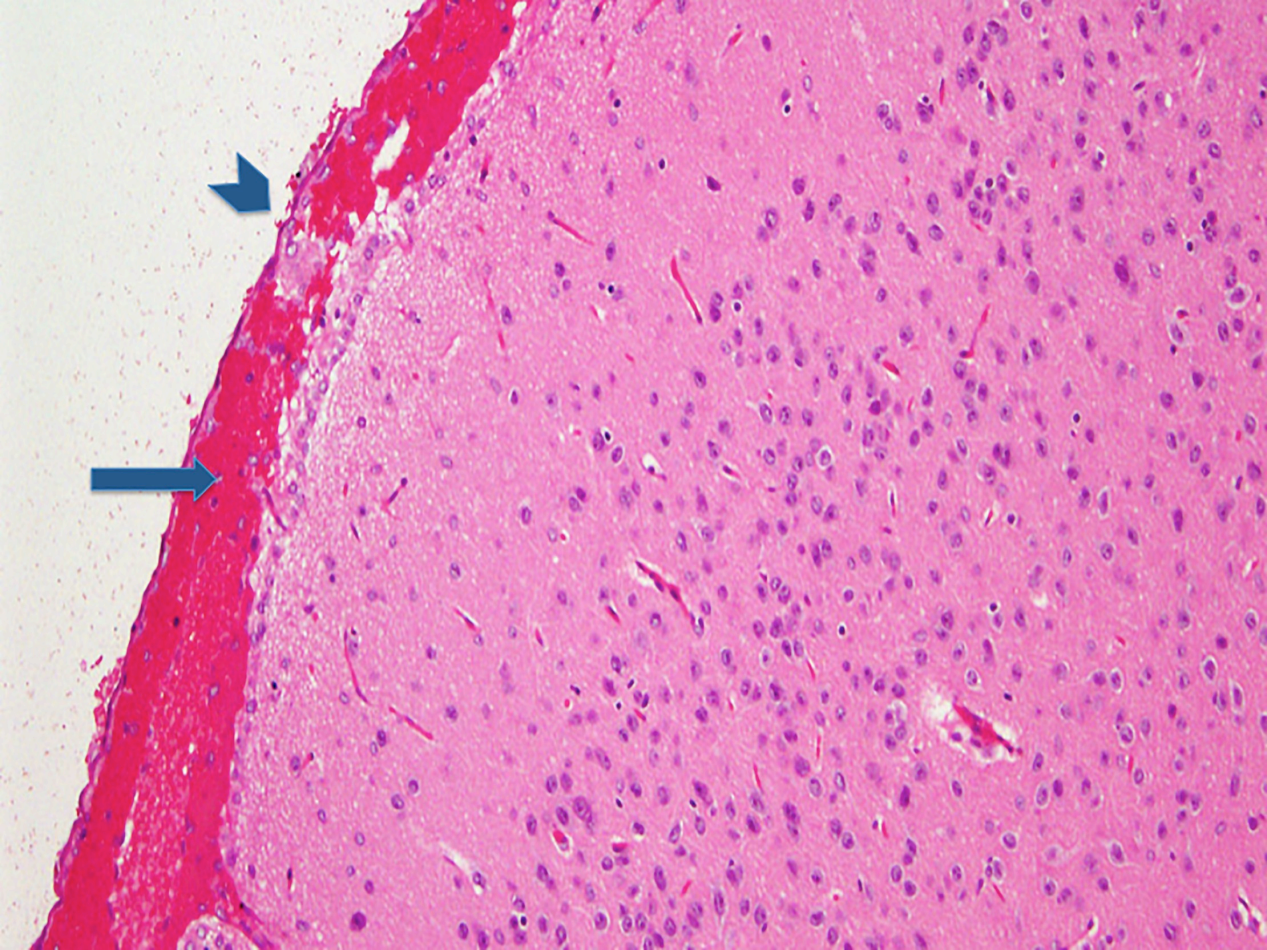
Grade III: A section of brain surface showing the surrounding subarachnoid space with a thick local layer of subarachnoid blood visible within the subarachnoid space (short blue arrow pointing at the arachnoidea, long blue arrow pointing at the a thik blood layed within the subarachnoid space).

**Fig 4:**
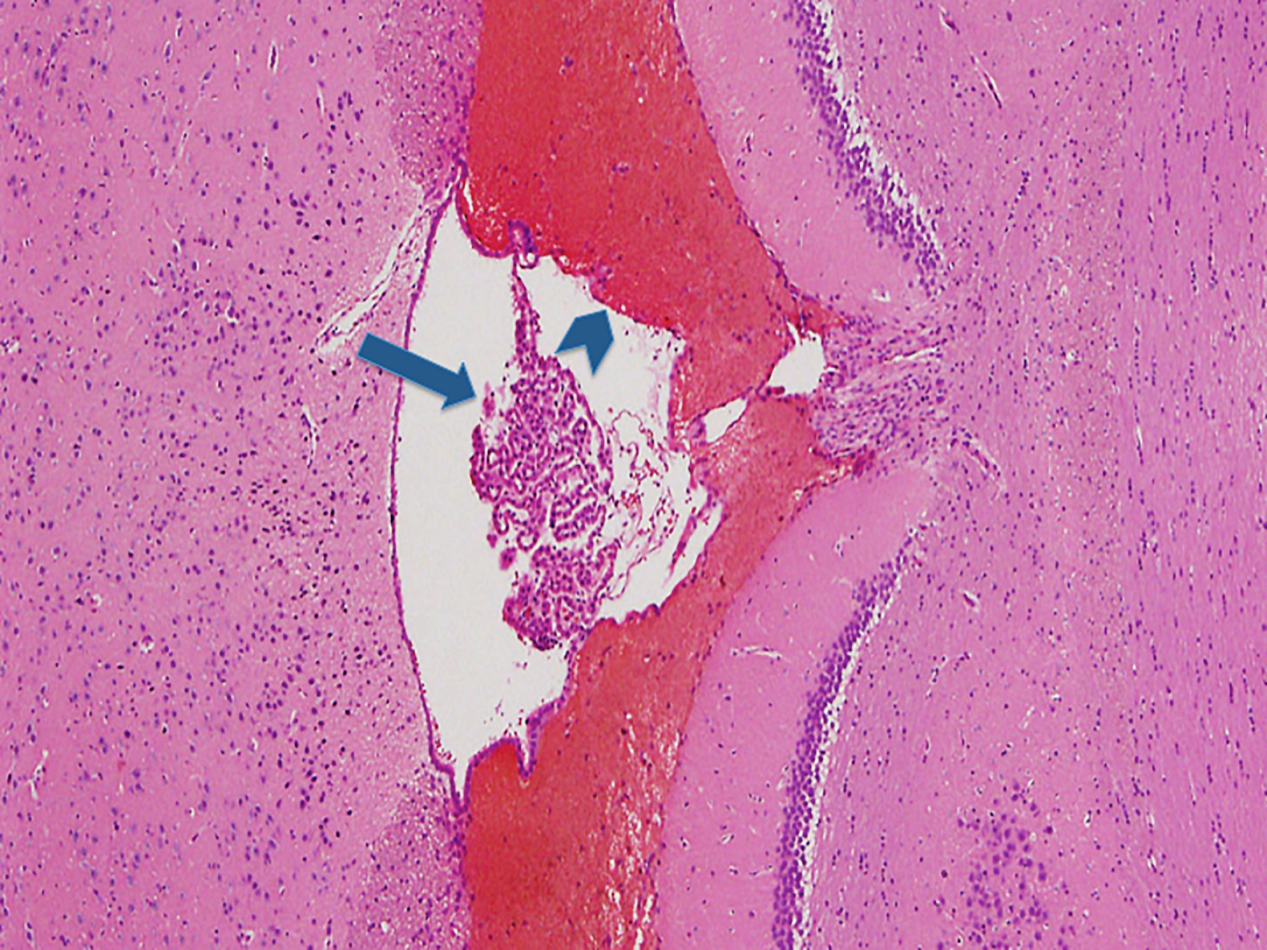
Grade IV: Section of the fourt ventricle with the choroidal plexus and diffuse blood clot visible within the ventricular cavity (long blue arrow pointing at the coroidal pexus and short blue arrow pointing at the blood clot within the ventricular cavity of the fourth ventricle).

The following experimental subarachnoid hemorrhage severity score (ESAS-score) was defined according to the histological findings (Table 1): grade I: no eSAH (sham group); grade II: local or diffuse thin eSAH, no IVH; grade III: diffuse / thick layers of blood, no IVH; grade IV: additional intraventricular hemorrhage. Grade I (Fig 1) was detected in 7.9% of eSAH rats (5/63), grade II (Fig 2) in 28.6% (18/63), grade III (Fig 3) in 41.3% (26/63) and grade IV (Fig 4) in 22.2% (14/63) of the rats with eSAH.

**Table 1:** Experimental subarachnoid hemorrhage grading scale (ESAS-score).

Grade I	No blood visible within the subarachnoid space, the ventricles or brain parenchmya
Grade II	Local or diffuse thin subarachnoid blood, no intraventricular or intraparenchymal blood
Grade III	Diffuse or local thick layers of subarachnoid blood, no intraventricular or intraparenchymal blood
Grade IV	Subarachnoidal blood independent of thickness or location plus intraventricular or intraparenchymal blood

### Meningeal reaction

**Fig 5:**
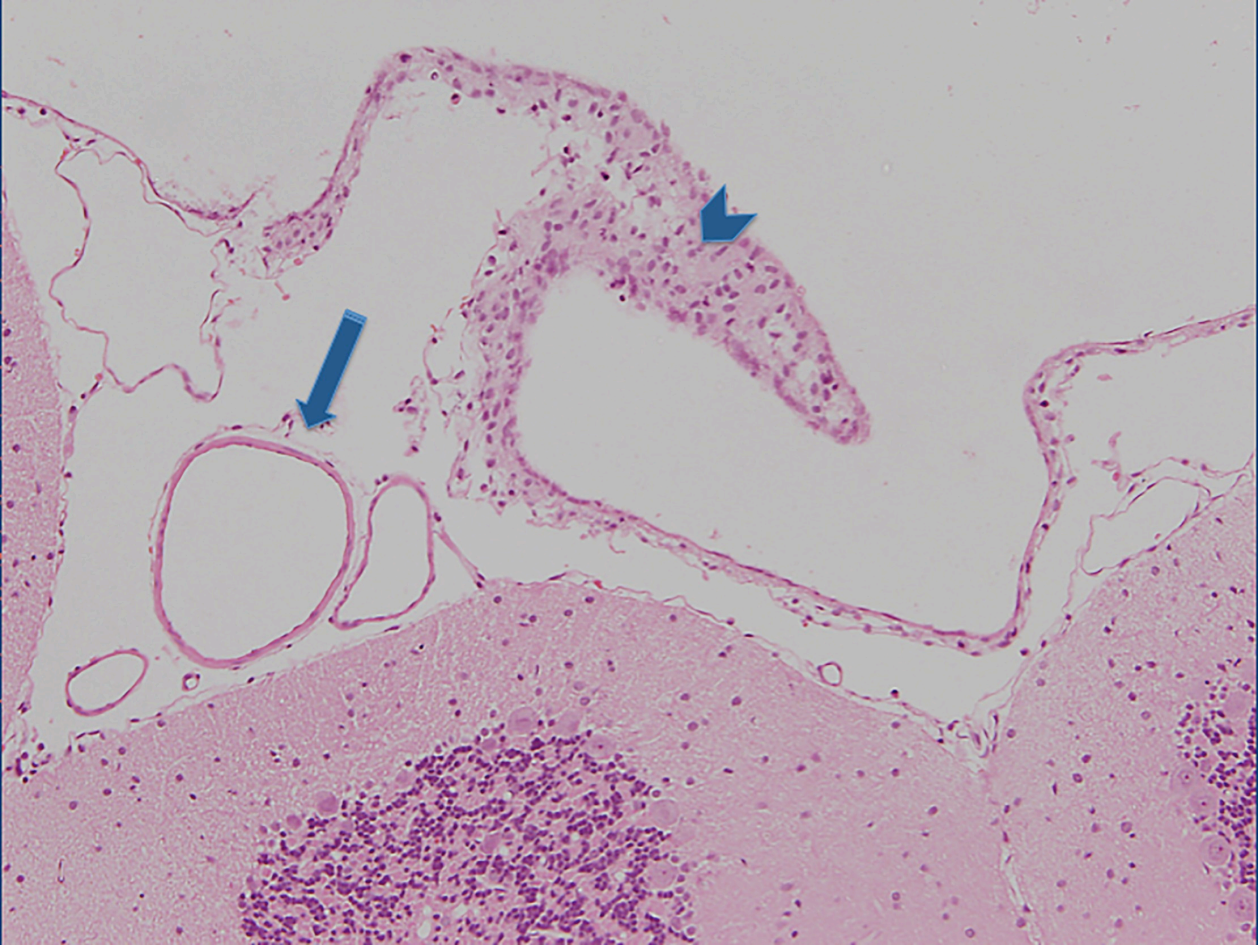
A section of brain surface showing the surrounding subarachnoid space with slight meningeal reaction and local mild meningeal cell proliferation induced by SAH (long blue arrow pointing at a vessel wihtin the subarachnoid space and short blue arrow pointing at the meninges with local meningeal cell proliferation).

**Fig 6:**
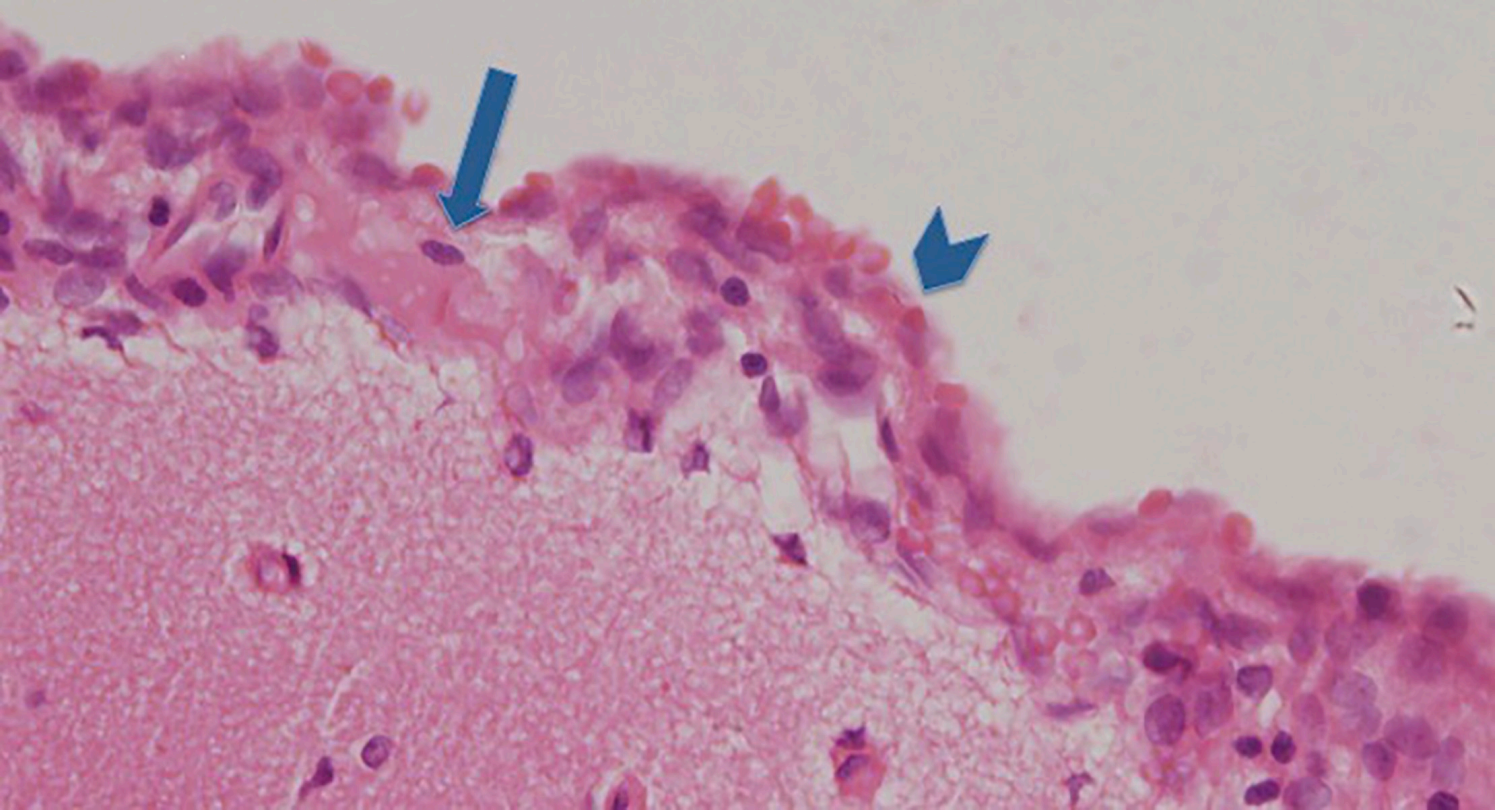
A section of brain surface showing the surrounding subarachnoid space with pronounced meningeal reaction and diffuse severe meningeal cell proliferation induced by SAH (short blue arrow pointing at the arachnoidea and long blue arrow pointing at diffuse meningeal cell proliferation within the subarachnoid space).

**Fig 7:**
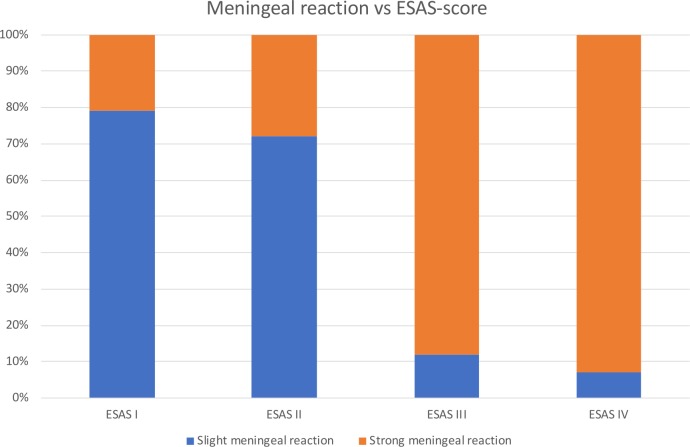
Meningeal reaction versus ESAS-score.

Furthermore, we found a meningeal reaction to the subarachnoid blood in all eSAH rats. We were able to distinguish between two different types of presentation. The meninges showed cell proliferation, which was graded either slight or pronounced. In 33.3% we saw a slight reaction (21/63) and in 66.7% (42/63) a pronounced cell proliferation (Figs 5 and 6). In all sham cases (8/8) there was no meningeal reaction. In 4 out of 5 rats with ESAS-score I a slight meningeal reaction was seen and in 1 rat a pronounced meningeal reaction was detected. We found a statistically significant correlation of a high ESAS-score with a pronounced meningeal reaction (Spearman’s correlation coefficient 0.652, *p*<0.0001). Fig 7 shows the meningeal reaction in the different ESAS-score groups.

### Neuroscore

**Fig 8:**
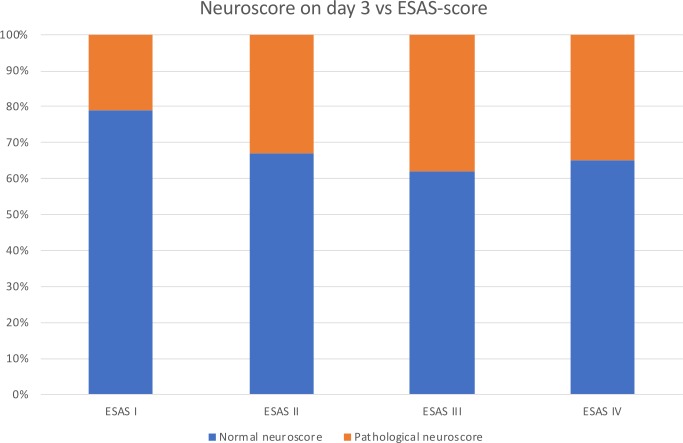
Neuroscore versus ESAS-score.

On day 3, a pathological neuroscore was found in 22/63 eSAH-rats (34.9%). Only one rat (1/22, 4.7%) had a high neuroscore of 3, whereas 6 rats (6/22, 27.2%) had a neuroscore of 2 and 15 rats (15/22, 68.1%) a neuroscore of 1. In none of the rats, a neuroscore of 4 was found. In 68.1% (15/22) of the rats with pathological neuroscore a high ESAS-score (III-IV) was present. In 15/40 rats (37.5%) with a high-grade eSAH (ESAS III and IV), a pathological neuroscore was found on day 3, compared to 6/23 rats (26%) with a low-grade eSAH (ESAS I and II). The distribution of a pathological neuroscore within the different ESAS-score rat groups is shown in Fig 8. There was no significant correlation of a pathological neuroscore on day 3 and a high ESAS-score (Spearman’s correlation coefficient 0.115, *p* = 0.368). On day 4, only 7/63 rats had a pathological neuroscore (11.1%) and on day 5 the neuroscore was pathological in 4/63 rats (6.3%). In 5/7 (71.4%) of the rats with a pathological neuroscore on day 4, a high ESAS-score was present. All 4 rats with a pathological neuroscore on day 5 had a high ESAS-score.

### Report on adverse events

The overall mortality rate was 49.6% (71/143) including the sham-group and 46.6% with regards to the eSAH-group (63/135).

## Discussion

In this experimental study, we developed a grading system for the distribution and amount of blood within the subarachnoid space and the ventricular system in the double hemorrhage SAH model in rats based on post mortem histological analysis, according to the well-established Fisher score in humans, which is based on the amount of blood in the subarachnoid space and its distribution within the ventricular system on the initial computed tomography scan [21]. Grade 3 and 4 of the Fisher score are considered high-grade SAH with a poor outcome in up to 74% of the cases [22]. With regards to the ESAS-score, we demonstrated that the double hemorrhage eSAH-model induces a high-grade eSAH (ESAS III and IV) in more than 60% of all rats, making it especially suitable for the evaluation of outcome parameters in severe SAH.

### Meeting different requirements on experimental SAH-models in rodents with respect to a different research focus

Experimental animal models help in the understanding of pathophysiological mechanisms, the evaluation of diagnostic as well as treatment options concerning specific diseases. In order to generate clinically applicable results, SAH-models have to reflect the clinical condition in humans as accurately as possible with the aim to facilitate a reliable translation from bench to bedside. No SAH-model currently exists, that would allow the assessment of all pathophysiological processes arising after SAH. The perforation SAH-model represents more realistically the scenario of aneurysm rupture in humans. Hence, the perforation SAH-model is considered more suitable for the assessment of the early processes occurring after SAH, which are summarized as early brain injury [5,8,10]. The perforation model, however, seems to be inferior for investigations with a focus on delayed cerebral ischemia (DCI) and vasospasm. The perforation SAH-model is associated with a relatively high variability of the SAH severity and mortality (15–50%) due to a lack of control over the amount of extravasated blood after the vessel perforation [7,8]. However, the low incidence of vasospasm (16.5%) suggests a comparatively low volume of cisternal blood after artery perforation. In contrast to that, the blood injection SAH-models do not realistically reproduce the aneurysm rupture in humans but ensure better control of the injected amount of blood. The often higher amount of cisternal blood makes the blood injection models more appropriate for the investigation of DCI and vasospasm [10,12,18].

The incidence of vasospasm is as high as 33.3%, if the double blood injection eSAH-model is used [5]. The mortality in the blood injection models is higher than in the perforation models, outlining again the association of the amount of cisternal blood, severity of SAH and outcome. Depending on the research focus, the most suitable SAH-model has to be selected, taking the different mortality rates of the perforation and the blood injection models into account.

### Grading systems of SAH-severity

Clinical and radiological grading scores are well established to define the severity of SAH in humans. These grading scores are used to classify the patient’s initial clinical condition after the bleeding [23,24] or the distribution and amount of blood [21,23, 25–27] within the subarachnoid space, ventricular system and brain parenchyma. Most often, these grading systems are used to prognosticate the patient’s outcome. Furthermore, an association between the severity of SAH, the amount of subarachnoid blood and incidence of DCI and vasospasm was shown.

The establishment of grading systems in SAH models would improve the interpretation of the experimental results within each study and allow a more sophisticated comparison of studies using different eSAH-models. For the transfer of the experimental data into the clinical setting, a certain similarity of the grading systems in patients and experimental animals would be helpful. The development of a clinical grading system in rodents, which resembles that of humans, is challenging because many symptoms in humans are not reproducible in small animals. Therefore, we and others focussed on the development of a grading system, which assesses the amount of intracranial blood. The intracranial blood can be depicted by magnetic resonance imaging [16,28]. While conventional and widely available MRI scanners with 1.5 T or 3 T does not allow a sufficient visualization of blood within the subarachnoid space and ventricular system in eSAH-models in rats, 7 Tesla MRI depicts intracisternal blood. Shishido et al. have proposed a 7 Tesla MRI-based grading system for eSAH according to the Claasen score in humans. Using the perforation model, they found a strong correlation of the MRI grading scale with the post mortem SAH grading as well as with the neurological score [15].

Unfortunately, 7 Tesla MR scanner are not widely available, reducing the use of that radiological grading system of eSAH. The possibility of repetition of imaging at different time points appears as the greatest advantage of small animal MRI. However, as in the clinical and radiological grading systems in humans, the definition of the time point of examination is necessary to guarantee for interindividual comparability. While in humans, the best and commonly used time point for grading is shortly after aneurysm rupture, the best time point appears being related to the focus of the experiment, with early imaging if the evaluation of early brain injury is of interest and late imaging, if DCI and vasospasm are investigated.

Intracisternal blood can also be identified either by post mortem analysis by direct macroscopic visualization or by histological analysis of the removed brain [10]. The wide availability of histological analysis using the HE (hematoxylin eosin)-staining is an advantage of post mortem histology-based evaluation of eSAH-severity and was used for the proposed ESAS-score. The possible variation of the histological findings, depending on the time point of examination in relation to the SAH induction, has to be taken into account, with possibly lower scores later after induction of eSAH. In our study, in 5 out of 63 rats with eSAH, no blood was visible in the histological sections. Since the histological analyses were performed on day 5 after the SAH induction in this study, we cannot exclude a hematoma resolution in these cases. However, the same has to be taken into account if using the MRI grading scale not immediately after induction of the eSAH. A limitation of the introduced ESAS-score is its semiquantitative and not quantitative manner. Nevertheless, high amounts of blood within the subarachnoid space and/or ventricular system could be detected in more than 60% of the SAH-rats. Furthermore, a pronounced meningeal reaction was significantly more often found in the rats with a high ESAS-score compared to the rats with a low ESAS-score, which supports the results of the proposed ESAS-score. Although the evaluation of a meningeal reaction was performed according to clearly pre-defined criteria, qualitative instead of quantitative criteria were applied, which can be considered being a limitation of this study.

In contrast to the study of Shishido et al., we found no correlation of the ESAS-score with the neuroscore, which might be explained by the application of different neuroscores.

The double blood injection model allows the induction of a severe eSAH, that can be reliably scored using the ESAS-score. Additionally, the double blood injection eSAH-model is associated with a calculable mortality rate [4,12,18,20]. These findings might contribute to more reliable planning of future experimental studies of severe SAH with the possibility to reduce the number of animals included. It still remains unclear, if the introduced ESAS-score is reliably applicable to other eSAH-models. Hence, further studies are needed to evaluate this new grading score in different eSAH-models and the validity of its applicability. A comparison of the ESAS-score with an MRI-based grading system might contribute to the evaluation of its applicability.

## Conclusions

The modified double hemorrhage model allows the induction of a severe (high grade) SAH in more than 60% of all rats, making it especially suitable for the evaluation of outcome parameters or DCI/vasospasm experiments.

According to the results of this study, the ESAS score seems to be a reliable tool for the assessment of eSAH severity. The reliability of the ESAS score for the evaluation of the eSAH severity has to be evaluated in a separate study by an additional macroscopical assessment of the eSAH severity by sacrificing the animals direct after the SAH induction. Furthermore, we recommend to validate the ESAS-score in other SAH-models in order to better evaluate experimental findings and optimize the comparability of studies.

## Compliance with ethical standards

All applicable international, national and institutional guidelines for the care and use of animals were followed. All experiments were conducted in accordance with the “Guide for the Care and Use of Laboratory Animals of the NIH”and were ethically approved by the Government of Lower Saxony. The approval number was AZ 13/1055. Anesthesia was performed by intraperitoneal injection of an anesthetic cocktail (1 ml) consisting of 0.3 ml medetomidine (Cepetor® 1 mg/ml) and 0.7 ml ketamine (Ketamin 10% Solution) with an applied dosage of 0.1 ml per 100 g body weight. Euthanasia was performed by transcardial perfusion under general anesthesia as described above.
